# Immune Effector Cell-Associated Hemophagocytic Lymphohistiocytosis Following CAR T-Cell Therapy: Results of a Real-World Study

**DOI:** 10.3390/cancers18101594

**Published:** 2026-05-14

**Authors:** Inna Shaforostova, Marie-Noelle Kronig, Katja Seipel, Alicia Rovo, Ulrike Bacher, Thomas Pabst

**Affiliations:** 1 Department of Medical Oncology, Inselspital Bern University Hospital, University of Bern, 3010 Bern, Switzerland; marie-noelle.kronig@insel.ch; 2 Department for Biomedical Research, University of Bern, 3010 Bern, Switzerland; katja.seipel@unibe.ch; 3 Department of Hematology and Central Hematology Laboratory, Inselspital Bern University Hospital, University of Bern, 3010 Bern, Switzerland; alicia.rovo@insel.ch (A.R.); veraulrike.bacher@insel.ch (U.B.)

**Keywords:** CAR T-cell therapy, IEC-HS, ASTCT, CRS

## Abstract

**Background**: Immune effector cell-associated hemophagocytic lymphohistiocytosis-like syndrome (IEC-HS) is a rare, life-threatening complication following CAR T-cell therapy. Diagnosis is challenging due to overlap with severe CRS, sepsis and lack of standardized criteria. Clinical data remain limited. **Methods**: We retrospectively analyzed 301 patients treated with CD19- or BCMA-directed CAR T-cells for hematologic malignancies at a single center from January 2019 to January 2026. IEC-HS was defined according to American Society for Transplantation and Cellular Therapy criteria. **Results**: Median follow-up was 31 months. IEC-HS was diagnosed in 14 patients (4.7%), median age 67 years. Underlying diseases included diffuse large B-cell lymphoma (*n* = 4), multiple myeloma (*n* = 7), mantle cell lymphoma, Burkitt lymphoma and B-lymphoblastic leukemia (*n* = 1 each). All patients had hyperferritinemia and cytopenias at baseline; most had high tumor burden (9/14) and elevated LDH (10/14). CRS occurred in all patients and ICANS in 6/14. IEC-HS occurred at median 10 days and was characterized by hyperferritinemia (median 15,321 µg/L), neutropenia, thrombocytopenia, hepatic dysfunction and high CAR-T-cell expansion in peripheral blood. Treatment included corticosteroids and anakinra (12/14). Refractory patients received IVIG (5/14), tocilizumab (3/14), siltuximab, ruxolitinib, emapalumab or etoposide (each *n* = 1). Infections occurred in 11/14; 4/14 had mixed infections. IEC-HS resolved in 7/14 (median 7 days). Mortality was 79% (11/14), mainly due to IEC-HS (7/14). Three patients were alive at last follow-up. One-year OS was lower vs. the whole cohort (31% vs. 69%, *p* < 0.0001). **Conclusions**: IEC-HS was associated with severe cytopenias, hyperferritinemia, hepatic dysfunction and high infection risk. Despite intensive immunosuppressive therapy, outcomes remain poor. Early biomarker-driven identification and multicenter studies are needed.

## Simple Summary

Immune effector cell-associated hemophagocytic lymphohistiocytosis (IEC-HS) is a rare but potentially fatal complication following CAR T-cell therapy that differs from other types of secondary hemophagocytic lymphohistiocytosis. Evidence remains limited due to the low incidence, lack of uniformly accepted diagnostic criteria and the considerable overlap with cytokine release syndrome and severe infection. In this study, we analyzed the incidence, risk factors, management and outcomes of IEC-HS in patients with B-cell lymphomas and multiple myeloma. Diagnosis was established according to the ASTCT consensus criteria. IEC-HS was associated with high tumor burden, elevated ferritin, cytopenia and higher CAR T-cell expansion. Mortality was high despite treatment mostly including anakinra and steroids. Prospective multicentric studies are required to refine diagnostic criteria, identify early laboratory markers and optimize the treatment strategies.

## 1. Introduction

Chimeric antigen receptor (CAR) T-cell therapy has markedly transformed the therapeutic landscape and prognosis of patients with various B-cell malignancies, including B-cell lymphomas, B-cell acute lymphoblastic leukemia (B-ALL), and multiple myeloma (MM) [1,2,3]. In addition to its established clinical efficacy, CAR T-cell therapy is associated with unique immune-related toxicities, most notably cytokine release syndrome (CRS) and immune effector cell-associated neurotoxicity syndrome (ICANS) [4,5].

A less common yet potentially life-threatening immune-related complication after CAR T-cell therapy is hemophagocytic lymphohistiocytosis (HLH). HLH is a hyperinflammatory syndrome characterized by excessive activation of macrophages and lymphocytes, leading to overproduction of proinflammatory cytokines, hemophagocytosis, and multiorgan dysfunction [6]. Within the context of CAR T-cell therapy, this condition is most commonly termed immune effector cell-associated hemophagocytic lymphohistiocytosis-like syndrome (IEC-HS). It represents a distinct clinical entity that shares overlapping features with secondary HLH. However, fulfillment of all conventional HLH diagnostic criteria is not required to establish the diagnosis and initiate treatment [7]. The incidence of IEC-HS is reported to be between 2.7% and 14.8% for CD19 or BCMA (B-cell maturation antigen-based) CAR T-cell therapies [7,8].

The pathophysiology of IEC-HS remains to be fully elucidated. Proposed mechanisms include interferon-gamma (INF-γ) mediated macrophage activation via signaling initiated by CAR T-cells and the release of damage-associated molecular patterns from tumor cells resulting in uncontrolled macrophage activation and proliferation, leading to tissue injury and multiorgan dysfunction [9].

The HLH-2004 diagnostic criteria are commonly employed for the diagnosis of both primary and secondary HLH. However, recent evidence indicates that these criteria may not be fully applicable to IEC-HS. Consequently, alternative criteria systems have been developed to enhance diagnostic accuracy and clinical recognition in this specific context. The most prominent of these are the carHLH criteria proposed by the CARTOX Working Group [10] and the IEC-HS criteria, developed by the American Society for Transplantation and Cellular Therapy (ASTCT) [11].

The most consistently reported risk factor for IEC-HS is the presence of preceding cytokine release syndrome (CRS). Other contributing factors may include a high baseline tumor burden, markedly elevated serum ferritin levels prior to IEC-HS onset, prolonged duration of CRS, robust CAR T-cell expansion and recent or concurrent infections [12,13].

Given the potentially fulminant course of IEC-HS, timely recognition and initiation of therapy are critical. At present, there is no accepted standard of care for IEC-HS; management is generally extrapolated from strategies used for secondary HLH. Systemic corticosteroids remain the cornerstone of therapy and are usually combined with additional anti-inflammatory agents, such as the interleukin-1 receptor antagonist anakinra. Other agents including intravenous immunoglobulin (IVIG), JAK1/2 inhibitors (e.g., ruxolitinib) and interferon-γ (IFN-γ) antagonists (e.g., emapalumab) may be used in refractory cases of IEC-HS [12,14,15,16].

Although IEC-HS is now recognized as a distinct toxicity associated with CAR T-cell therapy, the current understanding of its pathophysiology, clinical presentation, and optimal management remains limited. Much of the existing literature consists of case reports and small case series [8,15,17,18,19]. Therefore, we conducted a retrospective study aimed at characterizing the incidence, diagnostic criteria, clinical features, and management strategies of IEC-HS observed in patients with hematological malignancies following CAR T-cell therapies at our institution, the largest tertiary center in Switzerland.

## 2. Materials and Methods

A single-center retrospective study was conducted at the University Hospital Bern, assessing all consecutive patients with hematological malignancies who developed immune effector cell-associated hemophagocytic lymphohistiocytosis following CAR T-cell therapy with idecabtagene vicleucel, brexucabtagene autoleucel, lisocabtagene maraleucel, axicabtagene ciloleucel, tisagenlecleucel, and ciltacabtagene autoleucel during the period from January 2019 to January 2026. Only commercial products were included. Patient data were obtained from electronic health records and laboratory databases. Collected variables included demographic data, laboratory parameters prior to lymphodepletion therapy and at the time of IEC-HS diagnosis, including CAR T-cell activity as measured by digital droplet PCR (ddPCR) [20], as well as details regarding HLH-specific treatment interventions. CAR T-associated toxicities were evaluated in accordance with the ASTCT 2019 consensus guidelines [5].

The diagnosis of IEC-HS was established according to the modified ASTCT criteria proposed by Hines et al. in 2023 [11]. In patients treated between 2019 and 2023, the IEC-HS diagnosis was re-evaluated according to the ASTCT criteria, and only patients who fulfilled the criteria were included [11]. High tumor volume was defined separately for different entities: for DLBCL and MCL, if a single lymphoma manifestation was >7.5 cm; for multiple myeloma, if bone marrow infiltration >50% was present; for B-ALL, if >20% blasts in the bone marrow were present [21,22,23]. Treatment response in IEC-HS was defined by a reduction or normalization of ferritin levels, liver enzymes, and fibrinogen, in addition to improvement of cytopenias and overall clinical status.

Baseline characteristics were summarized using descriptive statistics. *p*-values were calculated using the Wisconsin test for continuous variables and Fisher’s exact test for categorical variables. Overall survival (OS) was defined as the time from CAR T-cell infusion to death from any cause. OS was estimated using the Kaplan–Meier method, with comparisons between groups performed using the log-rank test. Follow-up time was calculated using the reverse Kaplan–Meier method. A two-sided *p*-value <0.05 was considered statistically significant. All statistical analyses were performed using R software, version 4.4.2 (2024).

Written informed consent was obtained from all patients, and the study adhered to the principles of the Declaration of Helsinki. The study was approved by the local ethics committee of Berne, Switzerland (decision number 2025-01905, date 23 September 2025).

## 3. Results

### 3.1. Patient Characteristics

Among 301 patients treated with CAR T-cell therapy at the University Hospital Bern, 14 patients (4.7%) developed IEC-HS. The patient characteristics are summarized in Table 1 and Supplementary Table S1. The median age at the time of CAR T-cell infusion was 67 years (range: 39–82), with a male predominance (11/14 patients, 79%). Underlying malignancies included multiple myeloma (*n* = 7), diffuse large B-cell lymphoma (DLBCL, *n* = 4), B-acute lymphoblastic leukemia (B-ALL, *n* = 1), mantle cell lymphoma (MCL, *n* = 1) and Burkitt lymphoma (BL, *n* = 1). All patients with B-cell lymphoma presented with advanced-stage disease, while four of six MM patients had stage I disease at diagnosis based on the Revised International Staging System (R-ISS).

Patients had received a median of three prior treatment lines (range: 2–6). High-dose chemotherapy followed by autologous stem cell transplantation (HDCT/ASCT) was performed in seven patients, including five with MM, one with DLBCL, and one with MCL. Bridging therapy was administered in eleven patients (79%), with treatment regimens selected based on disease type and clinical context.

Relapsed or refractory disease was present in 9/14 patients (64%), and 8/14 patients (57%) had a high tumor burden at the time of lymphodepletion.

CAR T-cell products administered were as follows: ciltacabtagene autoleucel (cilta-cel, *n* = 4), idecabtagene vicleucel (ide-cel, *n* = 3), brexucabtagene autoleucel (brexu-cel, *n* = 2), lisocabtagene maraleucel (liso-cel, *n* = 1), axicabtagene ciloleucel (axi-cel, *n* = 3), and tisagenlecleucel (tisa-cel, *n* = 1).

### 3.2. Laboratory Parameters Prior to Lymphodepletion

All patients exhibited markedly elevated ferritin levels prior to lymphodepletion, with a median value of 1584 µg/L (range: 544–6158 µg/L). Elevated lactate dehydrogenase (LDH) levels were observed in nine patients (64%), with a median of 316 U/L (range: 154–1137 U/L). Anemia was universally present, with a median hemoglobin level of 86 g/L (range: 61–113 g/L). Thrombocytopenia was identified in nine patients (64%), with a median platelet count of 110 × 10^9^/L (range: 3–370 × 10^9^/L). Neutropenia was diagnosed in three patients (median absolute neutrophil count 2.1 G/L (range: 0.01–11.38 G/L)), as shown in Supplementary Table S2.

### 3.3. Toxicities After CAR T-Cell Infusion

All patients developed CRS, with three cases (21%) reaching grade ≥ 3. The median time to CRS onset was day one post-infusion (range: 0–9), and the median duration was three days (range: 2–10). ICANS occurred in six patients (43%), including four patients with grade ≥ 3 neurotoxicity. The median time to ICANS onset was ten days post-infusion (range: 1–29), with a median duration of three days (range: 2–10 days) (Table 2 and Table S2).

### 3.4. IEC-HS: Clinical Features, Laboratory Parameters and Management

IEC-HS was diagnosed at a median of ten days post-CAR T-cell infusion (range 4–38 days). In six patients, IEC-HS followed ongoing or resolving CRS, while in eight patients, CRS had fully resolved prior to the onset of IEC-HS.

All patients demonstrated marked hyperferritinemia (median ferritin: 13,722 ng/mL, range: 7182–101,815 ng/mL) and hypofibrinogenemia (median fibrinogen: 0.77 g/L, range: 0.41–1.51 g/L). Cytopenia was observed in all patients, including grade IV neutropenia and grade IV thrombocytopenia in eleven patients each (median platelet count 5 × 10^9^/L; range 2–51^9^/L; median neutrophil count 0.1 × 10^9^ G/L; range: 0.01–1.88^9^ G/L). Anemia was diagnosed in all patients with a median hemoglobin level of 69 g/L (range: 60–75 g/L). Hepatic dysfunction, defined by elevated transaminases and/or bilirubin, was observed in twelve patients (86%), with grade four liver toxicity in three cases. Hypertriglyceridemia was present in all evaluable patients (median triglyceride level: 5.03 mmol/L, range: 1.39–12.96 mmol/L) (Supplementary Table S2).

All patients showed a marked expansion of CAR T cells, with a median of 54,806 copies/mL (range: 5662–390,196 copies/mL) (Table 2).

Corticosteroids were administered to twelve patients (median duration: 15 days, range: 4–36 days), with a median cumulative dose of 724 mg (range: 150–1430 mg); dexamethasone was used in all cases. Anakinra was used in twelve patients (median duration 12 days (range: 2–48 days), at a dosage of 100–200 mg twice daily). In eleven patients, dexamethasone and anakinra were combined. IVIG was administered in five patients (29%). Four patients received tocilizumab as part of IEC-HS therapy. In a few patients who were refractory to first-line IEC-HS treatment, other agents were used. Two patients received ruxolitinib, and one patient each received siltuximab, etoposide, and emapalumab. One patient, initially suspected of septic shock, did not receive HLH-directed therapy and was diagnosed with IEC-HS postmortem. Vasopressor support was required in six cases. The data are summarized in Table 3.

Infectious complications occurred in eleven patients (79%), the majority had bacterial infections (*n* = 10). Three patients developed cytomegalovirus (CMV) viremia, and two systemic fungal infections, including pulmonary aspergillosis and mucormycosis. In four patients, mixed infections were diagnosed.

### 3.5. IEC-HS: Treatment Outcomes

Resolution of IEC-HS occurred in seven patients (50%) after a median duration of seven days (range: 3–23 days). Early treatment response (day: 15–39 after CAR T-cell infusion) to CAR T-cell treatment was assessable in thirteen patients, with an overall response rate of 85% including six CRs, four PRs and one very good partial remission (VGPR) (Table 3).

**Figure 1:**
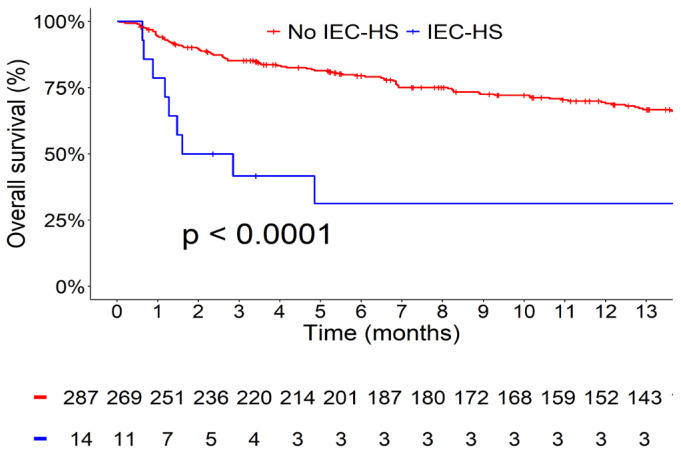
Overall survival in the IEC-HS group versus the overall CAR T-cell cohort. IEC-HS: Immune effector cell-associated lymphohistiocytosis-like syndrome.

One patient died prior to assessment. One-year survival was significantly lower in the IEC-HS group compared with the overall cohort without IEC-HS (31% [95% CI, 13–73] vs. 69% [95% CI, 64–75]; *p* < 0.0001; Figure 1).

The median follow-up was 29 months (range: 0.1–79 months). At the time of last follow-up, three patients (21%) remained alive, two with multiple myeloma and one with Burkitt lymphoma. Eleven patients died; the causes of death included active IEC-HS (*n* = 7), infections (*n* = 4), including one with ICANS (*n* = 1), late non-ICANS neurotoxicity (*n* = 1) and disease relapse (*n* = 1).

IEC-HS survivors more frequently had multiple myeloma and higher ferritin levels prior to lymphodepletion (*p* = 0.06 and *p* = 0.05, respectively). No differences regarding CRS or ICANS severity were observed. Patients with resolved IEC-HS showed a trend toward higher CAR T-cell expansion. No differences in the duration, type of treatment, or response to CAR T-cell therapy were found (Supplementary Table S3).

## 4. Discussion

This retrospective study represents one of the largest single-center analyses to date assessing the frequency, clinical characteristics, and outcomes of patients with IEC-HS treated with CAR T-cell therapy. The observed incidence of IEC-HS was 4.7%, consistent with previously reported rates in adult populations receiving CD19- or BCMA-directed CAR T-cell products [8,24].

The time to onset of IEC-HS was comparable to prior reports from the Mayo Clinic, and was earlier than typically observed in pediatric populations [8]. Notably, IEC-HS was associated with CRS in all cases, supporting previous observations that CRS represents a major risk factor for IEC-HS [12].

Several laboratory abnormalities prior to lymphodepletion therapy were identified as potential risk factors, including elevated LDH, thrombocytopenia, and anemia. LDH elevation may reflect high tumor burden, a known risk factor for IEC-HS [25]. Cytopenia has also been previously described as a risk factor and could serve as a surrogate marker of disease burden or bone marrow insufficiency resulting from lymphoma or myeloma infiltration, although toxicity from previous treatments is also possible [18,25,26]. These findings support the hypothesis that baseline disease activity contributes to the development of IEC-HS.

A markedly elevated ferritin level prior to IEC-HS onset represented another significant risk factor [7], and may indicate a pre-existing inflammatory state. Close monitoring of ferritin dynamics may therefore facilitate early identification of patients at high risk for IEC-HS and prompt initiation of treatment.

Another notable finding was profound CAR T-cell expansion in peripheral blood in all IEC-HS cases, consistent with previous reports [7,25] and suggesting that excessive immune activation may contribute both to treatment efficacy and to the development of hyperinflammatory toxicity [9].

Although genetic causes are common in primary HLH and testing is recommended in children and high-risk adults, no data support routine genetic testing in IEC-HS. IEC-HS is considered a distinct secondary inflammatory syndrome related to IEC therapy [27].

Given the rarity of IEC-HS, optimal management strategies remain uncertain. In our cohort, systemic corticosteroids and the interleukin-1 receptor antagonist anakinra were administered in accordance with recent recommendations [28,29]. In cases presenting with more severe clinical manifestations, high-dose intravenous immunoglobulin (IVIG) was employed, consistent with established guidelines for the management of hemophagocytic lymphohistiocytosis/macrophage activation syndrome (HLH/MAS) [26]. 

Other immunomodulatory agents, including siltuximab, etoposide, ruxolitinib and emapalumab, were utilized only occasionally and demonstrated no clear clinical benefit. The optimal treatment strategy for IEC-HS remains unknown. Data on treatment response are very limited because of the rarity of this condition and are largely extrapolated from general HLH treatment. Currently, first-line therapy for IEC-HS consists of dexamethasone with or without anakinra. While response rates to anakinra reach up to 90% in the general HLH population, data in IEC-HS remain unclear [30,31]. Second-line therapy includes ruxolitinib, with reported remission rates of up to 70% in pediatric non-IEC-HS cohorts [30]. Third-line options include emapalumab or low-dose etoposide. In a small case series, the response rate to emapalumab was 90% [31].

Consistent with prior studies, infectious complications were prevalent. These were predominantly bacterial bloodstream infections; however, fungal (e.g., mucormycosis) and viral (e.g., cytomegalovirus viremia) pathogens were also identified. The high rate of infections likely reflects the combined effects of prior therapies, CAR T-cell-related immune dysregulation, and immunosuppressive treatment for IEC-HS. These findings highlight the critical need for vigilant monitoring, prompt initiation of antimicrobial therapy, and consideration of prophylactic measures, particularly among patients undergoing prolonged immunosuppressive treatment for IEC-HS [12].

Overall mortality associated with IEC-HS remains high, consistent with previous reports highlighting the urgent need for earlier diagnosis and more effective therapy [7,8,18].

We analyzed differences between IEC-HS survivors and deceased patients. Multiple myeloma was associated with a more favorable clinical course compared with DLBCL. Higher CAR T-cell expansion was associated with improved survival; however, these differences did not reach statistical significance due to the small sample size. These findings suggest that patients with markedly elevated ferritin levels (median > 20,000) represent a high-risk IEC-HS subgroup associated with increased mortality. Also, we suggest that early initiation of combined high-dose steroids and high-dose anakinra (≥200 mg daily) may improve outcomes in this high-risk setting [27].

Our study has some limitations which include the retrospective design, the single-center study setting, and a small sample size. Due to the limited number of patients, individuals with different hematological malignancies and those treated with BCMA- and CD19-based CAR T-cell therapy were analyzed together, thereby reducing the statistical power of the study.

## 5. Conclusions

In conclusion, IEC-HS represents a rare but life-threatening complication of CAR T-cell therapy. Our real-world data contribute to the growing body of literature on this challenging clinical entity, particularly in the context of CD19- and BCMA-directed CAR T-cell therapies. Given the diagnostic complexity and substantial associated morbidity and mortality, early recognition and timely initiation of targeted immunosuppressive therapy are critical to improving patient outcomes. Elevated ferritin and LDH levels, as well as early and pronounced CAR T-cell expansion in the peripheral blood, may serve as potential biomarkers identifying patients at higher risk of developing IEC-HS. Future multicenter studies are warranted to further elucidate risk factors for IEC-HS, validate predictive scoring systems, and identify additional early and specific biomarkers. Future studies should focus on standardized diagnostic criteria, reliable biomarkers, and optimized treatment strategies and even consider evaluation of preemptive interventions to improve the safety of CAR T-cell therapies.

## Figures and Tables

**Table 1:** Baseline characteristics and laboratory results prior to lymphodepletion chemotherapy.

Patients	Result
Patients with IEC-HS, *n* (%)	14 (100%)
Age at CAR T-cell therapy, years, median (range)	67 (39–82)
Male sex, *n* (%)	11 (79%)
**Disease subtype**	
Multiple myeloma, *n* (%)	7 (50%)
Diffuse large B-cell lymphoma, *n* (%)	4 (29%)
B-cell acute lymphoblastic leukemia, *n* (%)	1 (7%) ^1^
Mantle cell lymphoma, *n* (%)	1 (7%)
Burkitt lymphoma, *n* (%)	1 (7%)
**Previous treatment**	
Prior lines of therapy, median (range)	3 (2–6)
Prior HDCT/ASCT, *n* (%)	7 (50%)
Bridging therapy, *n* (%)	11 (79%)
**Disease status at lymphodepletion**	
Complete remission, *n* (%)	1 (7%)
Partial remission, *n* (%)	1 (7%)
Stable disease, *n* (%)	3 (21%)
Progressive disease, *n* (%)	7 (50%)
Untreated relapse, *n* (%)	2 (14%)
Low tumor burden, *n* (%)	6 (43%)
High tumor burden, *n* (%)	8 (57%)
**CAR T-cell product**	
Ciltacabtagene autoleucel, *n* (%)	4 (29%)
Idecabtagene vicleucel, *n* (%)	3 (21%)
Axicabtagene ciloleucel, *n* (%)	3 (21%)
Brexucabtagene autoleucel, *n* (%)	2 (14%)
Lisocabtagene maraleucel, *n* (%)	1 (7%)
Tisagenlecleucel, *n* (%)	1 (7%)

ASCT, autologous stem cell transplantation; CAR, chimeric antigen receptor; HDCT, high-dose chemotherapy; IEC-HS, immune effector cell. ^1^ Cytogenetics: normal karyotype; FISH analysis negative. No prior allogeneic stem cell transplantation; two cycles of blinatumomab prior to CAR T-cell therapy.

**Table 2:** Toxicities, biomarker data and outcome post-CAR T-cell therapy.

Parameter	Result
**CRS**	
Any grade CRS, *n* (%)	14 (100%)
Grade ≥ 3, *n* (%)	3 (21%)
Median time to onset, days (range)	1 (0–9)
Median duration, days (range)	3 (2–10)
**ICANS**	
Any grade ICANS, *n* (%)	6 (43%)
ICANS grade ≥ 3, *n* (%)	4/6 (67%)
Median time to onset, days (range)	10 (1–29)
Median duration, days (range)	3 (2–10) ^1^
**IEC-HS**	
During ongoing CRS, *n* (%)	10 (4–38)
After CRS resolution, *n* (%)	6 (43%)
Time to onset of IEC-HS, days, median (range)	8 (57%)
IEC-HS resolved, *n* (%)	7 (50%)
Time to resolution of IEC-HS, days, median (range)	7 (3–23)
**Biomarker data and toxicities at onset of IEC-HS**	
CAR T-Cell activity, number of copies1, median	54,806 (5662–390,196) ^a^
Bone marrow with hemophagocytosis signs, *n* (%)	3/4 (75%) ^2^
**Organ dysfunction at onset of IEC-HS**	
Pulmonary dysfunction grade ≥ 2, *n* (%)	6 (43%)
Kidney dysfunction grade ≥ 2, *n* (%)	5 (36%)
Hepatic dysfunction, grade ≥ 2, *n* (%)	12 (86%)
Hepatic dysfunction grade 4, *n* (%)	3 (21%)

CAR T: CAR T-cell activity: chimeric antigen receptor T-cell activity; CRS: cytokine release syndrome; ICANS: immune effector cell-associated neurotoxicity syndrome; IEC-HS: immune effector cell-associated hemophagocytic lymphohistiocytosis. ^a^ Measured by digital droplet PCR (ddPCR). ^1^ Data missing in one patient. ^2^ Only four patients underwent bone marrow puncture.

**Table 3:** Management and outcome of IEC-HS.

Baseline Characteristics	Result
**Management of IEC-HS, *n* (%)**	13 (93%)
Corticosteroids, *n* (%)	12 (86%)
Cumulative steroid dose, mg, median (range)	724 (150–1430) ^1^
Duration of steroid therapy, days (range)	15 (4–36)
Anakinra, *n* (%)	12 (86%)
Cumulative anakinra dose, mg, median (range)	5050 (200–30,400)
Duration of anakinra therapy, days (range)	14 (2–48)
IVIG, *n* (%)	5 (29%)
Tocilizumab, *n* (%)	4 (29%) ^2^
Etoposide, *n* (%)	1 (7%)
Siltuximab, *n* (%)	1 (7%)
Ruxolitinib, *n* (%)	1 (7%)
Emapalumab, *n* (%)	1 (7%)
Vasopressor support, *n* (%)	6 (43%)
**Infectious complications**	
Any infection, *n* (%)	11 (79%)
Bacterial infections, *n* (%)	10 (71%)
CMV-Reactivation, *n* (%)	3 (21%)
Fungal infections	2 (14%)
(Mucormycosis, pulmonary aspergillosis), *n* (%)	
Mixed infections, *n* (%)	4 (29%)
**Outcomes**	
Response to CAR T-cell treatment, *n* (%)	13 (93%) ^3^
CR, *n* (%)	4 (29%)
VGPR, *n* (%)	1 (7%)
PR, *n* (%)	6 (43%)
SD, *n* (%)	1 (7%)
Not available	1 (7%)
Resolution of IEC-HS	7 (50%)
Median time to resolution of IEC-HS, median, days (range)	7 (3–23)
Alive at last follow-up, *n* (%)	3 (21%)
**Causes of death**	11 (79%)
IEC-HS, *n* (%)	7 (64%)
Relapse and bacterial infection (sepsis), *n* (%)	1 (9%)
Bacterial infection (sepsis) + ICANS, *n* (%)	1 (9%)
Late neurotoxicity and sepsis, *n* (%)	1 (9%)
Sepsis, *n* (%)	1 (9%)

CR: complete remission; ICANS: immune effector cell-associated neurotoxicity syndrome; IEC-HS: immune effector cell-associated hemophagocytic lymphohistiocytosis; IVIG: intravenous immunoglobulin; PR: partial remission; SD: stable disease; VGPR: very good partial remission. ^1^ Dexamethasone was used in all cases; two patients did not receive steroids; missing data in one patient. ^2^ Cases only reported when tocilizumab was administered due to IEC-HS. ^3^ Treatment response was assessable in 13 patients.

## Data Availability

The original contributions presented in this study are included in the article. Further inquiries can be directed to the corresponding author.

## Supplementary Materials

The following supporting information can be downloaded at: https://www.mdpi.com/article/10.3390/cancers18101594/s1, Table S1: Baseline disease-specific characteristics at diagnosis prior to lymphodepletion; Table S2: Laboratory parameters at lymphodepletion and at onset of IEC-HS; Table S3: Comparison between IEC-HS Survivors and Non-Survivors.
