# Supplementary material for: Immune Effector Cell-Associated Hemophagocytic Lymphohistiocytosis Following CAR T-Cell Therapy: Results of a Real-World Study
Source: Cancers (Basel). 2026 May 14;18(10):1594. doi: 10.3390/cancers18101594 (PMC13204677; doi:10.3390/cancers18101594)
Supplement: Supplementary file 1 [file cancers-18-01594-s001.zip › cancers-4271763-supplementary.pdf]

Supplementary Table S1. Baseline disease-specific characteristics at diagnosis prior to lymphodepletion.

Baseline characteristics	Number of patients, n (%)
**B-cell lymphoma subgroup (DLBCL, MCL, BL)**	
Stage IV disease	6/6 (100%)
CNS involvement	2/6 (33%)
Bone marrow involvement	2/4 (50%)
LDH > ULN	3/3 (100%)
**Multiple myeloma subgroup**	
R-ISS stage I	4/6 (66%)
High-risk cytogenetics	3/4 (75%)
LDH > ULN	1/5 (20%)

BL: Burkitt lymphoma; CNS: central nervous system; DLBCL: diffuse large B-cell lymphoma; LDH: lactate dehydrogenase; MCL: mantle cell lymphoma; R-ISS: revised international staging system; ULN: upper limit of normal.

Supplementary Table S2. Laboratory parameters at lymphodepletion and at onset of IEC-HS.

Characteristics	At lymphodepletion	At onset of IEC-HS
Ferritin, µg/L, median (range)	1584 (544–6158)	13,722 (7,182–101,815)
CRP, mg/L, median (range)	23 (3–135)	42 (1–214)
D-dimer, µg/L, median (range)	1489 (410–5282)	6,682 (567–80,000)
LDH, U/L, median (range)	341 (100–1552)	804 (356–2500)
IL-6, pg/mL, median (range)	12 (3–75)	11,030 (799–1,292,910)
Fibrinogen, g/L, median (range)	3.52 (1.88–7.02)	0.77 (0.41–1.51)
Triglycerides, mmol/L, median (range)	—	5.03 (1.39–12.96)
Hemoglobin, g/L, median (range)	86 (61–113)	69 (60–75)
ANC, G/L, median (range)	2.1 (0.01–11.38)	0.1 (0.01–1.88)

ANC: absolute neutrophil count; CRP: C-reactive protein; IL-6: interleukin-6; LDH, lactate dehydrogenase. Normal ranges: Ferritin <250 µg/L; CRP <5 mg/L; D-dimer <500 µg/L; LDH <250 U/L; IL-6 <7 pg/mL; Fibrinogen 1.8–4.0 g/L; Hemoglobin 135–168 g/L; ANC 1.60–7.40 G/L; Platelets 150–450 G/L.

Supplementary Table S3. Comparison between IEC-HS Survivors and Non-Survivors

Parameter	IEC-HS survivors	IEC-HS non-survivors	p-value
**Baseline characteristics**			
Disease type (MM vs. DLBCL)	5 vs. 0	2 vs.4	0.06
Age, years (median)	65	68	0.90
Prior lines of therapy, median	4	3	0.89
Disease status at lymphodepletion (CR+PR)	1	1	1.00
**Biomarker prior lymphodepletion**			
Ferritin, µg/L, median	1,800	1,004	0.05
CRP, mg/L, median	28	18	1.00
IL-6, pg/mL, median	8	12	1.00
LDH, U/L, median	451	337	0.80
Fibrinogen, g/L, median	4.5	2.53	1.00
D-dimers, µg/L, median	1,512	778	0.91
Hemoglobin, g/L, median	87	85	0.65
ANC, G/L, median	3.42	1.88	0.90
Platelets, G/L, median	80	140	0.85
Lymphocytes, G/L, median	0.42	0.72	1.00
**Toxicities and biomarker data at the time of IEC-HS**			
CRS grade	7	7	1.00
Time to onset of CRS, days, median	1	1	1.00
Time to onset of IEC-HS, days, median	5	31	0.15
Ferritin, µg/L, median	12,976	20,261	0.53
CRP, mg/L, median	15	68	0.18
D-dimers, µg/L, median	14,052	3,678	0.57
LDH, U/L, median	949	658	1.00
IL-6, pg/mL, median	7,539	44,811	0.70
Fibrinogen lowest, g/L, median	0.7	0.83	0.37
Triglycerides, mmol/L, median	4.8	5.63	0.44
Hemoglobin, g/L, median	68	69	0.95
ANC, G/L, median	1.97	0.45	0.90
Platelets, G/L, median	5	5	0.37
CAR T-cell expansion, copies, median	82,987	16,929	0.05
**Management of IEC-HS**			
Steroids, duration, days, median	16	12	0.95
Anakinra, duration, days, median	17	7	0.18

ANC, absolute neutrophil count; CAR T-cell expansion, chimeric antigen receptor T-cell expansion; CRP, C-reactive protein; CR, complete response/complete remission; CRS, cytokine release syndrome; DLBCL: diffuse large B-cell lymphoma; IEC-HS, hemophagocytic syndrome; IL-6, interleukin-6; LDH, lactate dehydrogenase; MM, multiple myeloma; PR, partial response/partial remission. Normal ranges: Ferritin <250 µg/L; CRP <5 mg/L; D-dimer <500 µg/L; LDH <250 U/L; IL-6 <7 pg/mL; fibrinogen 1.8–4.0 g/L; triglycerides <1.7 mmol/L; hemoglobin 135–168 g/L; ANC 1.60–7.40 G/L; platelets 150–450 G/L.
